# Update on Neoadjuvant and Adjuvant BRAF Inhibitors in Papillary Craniopharyngioma: A Systematic Review

**DOI:** 10.3390/cancers16203479

**Published:** 2024-10-14

**Authors:** Giulia Cossu, Daniele S. C. Ramsay, Roy T. Daniel, Ahmed El Cadhi, Luc Kerherve, Edouard Morlaix, Sayda A. Houidi, Clément Millot-Piccoli, Renan Chapon, Tuan Le Van, Catherine Cao, Walid Farah, Maxime Lleu, Olivier Baland, Jacques Beaurain, Jean Michel Petit, Brivaël Lemogne, Mahmoud Messerer, Moncef Berhouma

**Affiliations:** 1 Department of Neurosurgery, University Hospital of Lausanne and University of Lausanne, 1011 Lausanne, Switzerland; roy.daniel@chuv.ch (R.T.D.);; 2 Department of Neurosurgery, University Hospital of Dijon Bourgogne, 21000 Dijon, Francecatherine.cao@chu-dijon.fr (C.C.); walid.farah@chu-dijon.fr (W.F.); maxime.lleu@chu-dijon.fr (M.L.);; 3 Imperial Brain and Spine Initiative, London W2 1NY, UK; 4 Imperial College School of Medicine, London W2 1PG, UK; 5 Department of Endocrinology, University Hospital of Dijon Bourgogne, 21000 Dijon, France; 6 Department of Neuroradiology, University Hospital of Dijon Bourgogne, 21000 Dijon, France; 7 Functional and Molecular Imaging Team (CNRS 6302), Molecular Chemistry Institute (ICMUB), University of Burgundy, 21078 Dijon, France

**Keywords:** craniopharyngioma, BRAF, inhibitors, MEK, papillary, systematic review, targeted therapy

## Abstract

**Simple Summary:**

Recent research uncovered the BRAF mutation in papillary craniopharyngiomas, leading to new targeted treatments that may reduce the need for invasive procedures. A systematic review of 20 studies with 37 patients, treated mostly in the U.S., found that 18 patients received these drugs after surgery or radiation (adjuvant treatment), while 19 received them before surgery (neoadjuvant treatment). The common combination of a BRAF inhibitor with a MEK inhibitor significantly shrank tumours, with reductions of 70% to 100% in many cases, and up to 91% for those treated before surgery. Some patients required no further treatment afterward. However, questions remain about the optimal use of these drugs, including timing, combinations, and managing side effects. Despite these challenges, targeted therapies are promising in improving outcomes and quality of life for patients with this brain tumour, with future studies expected to refine their use.

**Abstract:**

**Background/Objectives**: The recent discovery of BRAF mutation in papillary craniopharyngiomas opened new avenues for targeted therapies to control tumour growth, decreasing the need for invasive treatments and relative complications. The aim of this systematic review was to summarize the recent scientific data dealing with the use of targeted therapies in papillary craniopharyngiomas, as adjuvant and neoadjuvant treatments. **Methods**: The PRISMA guidelines were followed with searches performed in Scopus, MEDLINE, and Embase, following a dedicated PICO approach. **Results**: We included 21 pertinent studies encompassing 53 patients: 26 patients received BRAF inhibitors (BRAFi) as adjuvant treatment, while 25 received them as neoadjuvant treatment. In the adjuvant setting, BRAFi were used to treat recurrent tumours after surgery or adjuvant radiation therapy. The most common regimen combined dabrafenib (BRAFi) with trametinib (MEK1 and 2 inhibitor) in 81% of cases. The mean treatment length was 8.8 months (range 1.6 to 28 months) and 32% were continuing BRAFi. A reduction of tumour volume variable from 24% to 100% was observed at cerebral MRI during treatment and volumetric reduction ≥80% was described in 64% of cases. Once the treatment was stopped, adjuvant treatments were performed to stabilize patients in remission in 11 cases (65%) or when a progression was detected in three cases (12%). In four cases no further therapies were administered (16%). Mean follow-up after the end of targeted therapy was 17.1 months. As neoadjuvant regimen, 36% of patients were treated with dabrafenib and trametinib with a near complete radiological response in all the cases with a mean treatment of 5.7 months. The neoadjuvant use of verumafenib (BRAFi) and cometinib (MEK1 inhibitor) induced a near complete response in 15 patients (94%), with a median volumetric reduction between 85% and 91%. Ten patients did not receive further treatments. Side effects varied among studies. The optimal timing, sequencing, and duration of treatment of these new therapies should be established. Moreover, questions remain about the choice of specific BRAF/MEK inhibitors, the optimal protocol of treatment, and the strategies for managing adverse events. **Conclusions**: Treatment is shifting to a wider multidisciplinary management, where a key role is played by targeted therapies, to improve outcomes and quality of life for patients with BRAF-mutated craniopharyngiomas. Future, larger comparative trials will optimize their protocol of use and integration into multimodal strategies of treatment.

## 1. Introduction

Craniopharyngiomas constitute a small proportion of cerebral tumours, with an incidence around 0.5–2.0 cases per million persons per year [1,2]. However, they have the highest morbidity and mortality rate among sellar tumours [3], with a high propensity for local aggressiveness and involvement of local structures including the optic chiasm, pituitary gland, and hypothalamus [4]. Adamantinomatous and papillary craniopharyngiomas were previously considered to be subtypes of craniopharyngioma. However, since the 2021 World Health Organisation (WHO) Classification of Tumours of the Central Nervous System (CNS) [5], these are now considered two separate entities, owing to their different epidemiology, clinical behaviour, and peculiar radiological features, along with mutually exclusive mutations and methylation profiles [6].

Papillary craniopharyngiomas account for the minority of cases, most commonly appearing in adulthood between the ages of 40 and 60 years. Genetically, they are characterized by the BRAF V600E mutation, which contributes to the activation of the MAPK cascade and promotes cellular proliferation and survival. This mutation was shown to be present in more than 90% of papillary craniopharyngioma and provided a potential avenue for the integration of targeted therapy into their multimodal management [7,8].

Managing these tumours has traditionally been challenging due to their proximity to critical brain structures, requiring a careful balance between effective tumour control and the preservation of neurological function. Surgery remains the mainstay of treatment, with specific and often complementary indications for transcranial and endoscopic endonasal approaches [9]. The principal aim is to perform a maximal but hypothalamic-sparing resection to reduce post-operative morbidity [9,10,11] and the anatomical tumour location; the relationship with critical adjacent structures, along with the experience of the surgical team should be taken into account in surgical planning to select the optimal approach [9]. A radical surgical treatment can be curative, but it may be complicated by endocrinological deficits and hypothalamic syndrome in as much as 90% of cases [12]. More conservative surgical resections are associated with a lower morbidity rate but a higher risk of recurrence, and adjuvant radiation therapy on the residual tumour is key to ensure remission [9,13,14]. Indeed, residual and recurrent tumours not amenable to surgical resection are managed with adjuvant stereotactic radiotherapy [14] but, particularly with young patients, radiotherapy can present a significant risk of long-term complications. Alternative strategies of treatment, with lower toxicity, are thus required.

Recent clinical applications of BRAF inhibitors (BRAFi) in other solid malignancies resulted in a paradigm shift in their treatment, with high degrees of response and limited adverse events [15,16]. As BRAF mutations in papillary craniopharyngiomas are also ubiquitous, targeted molecular therapy was considered as an alternative to surgery and radiotherapy.

The significant response with limited side effects prompted further investigation and since the first use in 2016 [17], several case reports and case-series have been published, confirming its potential role in controlling the disease. Furthermore, the possible use of BRAFi as neoadjuvant treatment was recently described [18]. This may offer an alternative pathway for patients who may not be candidates for a radical surgical resection as first line of treatment [19], as it may serve to reduce tumour bulk allowing for more aggressive resections for lesions located in areas associated with high post-operative morbidity, thus increasing the safety of craniopharyngioma management in experienced hands.

This systematic review aimed to examine the literature for published case-reports and case-series relating to the use of adjuvant and neoadjuvant targeted molecular therapy in the treatment of papillary craniopharyngiomas.

## 2. Materials and Methods

**Figure 1:**
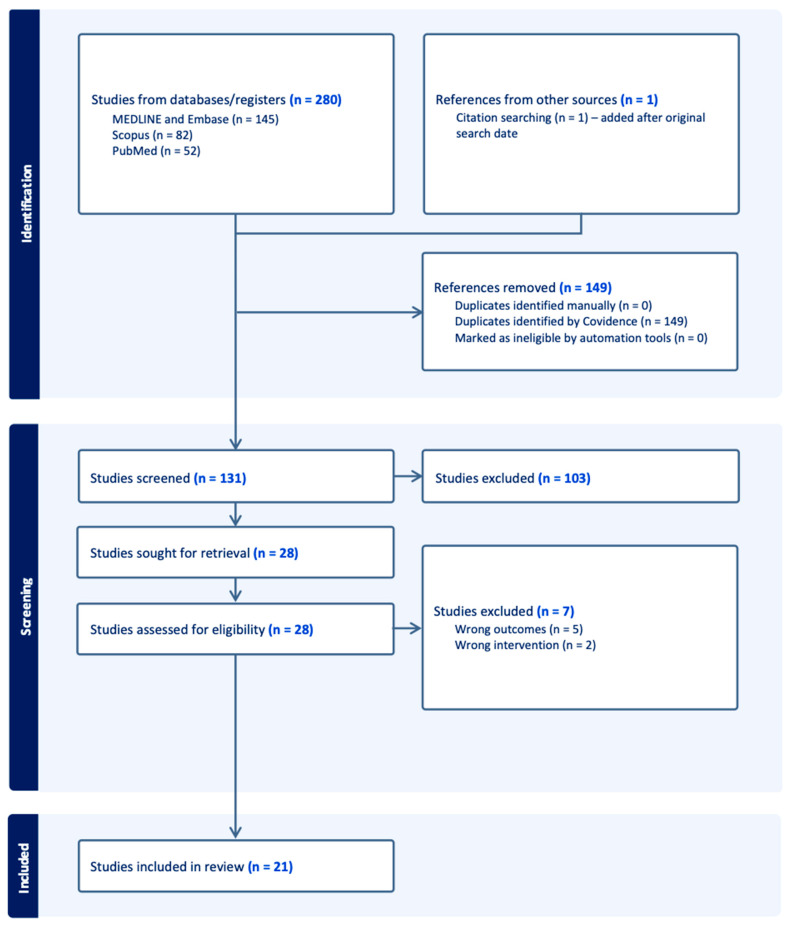
PRISMA flowchart demonstrating the results of database searches and the number of studies included during the screening process.

The Preferred Reporting Items for Systematic Reviews and Meta-Analyses (PRISMA) guidelines were followed when conducting this systematic review. Searches of academic databases including Scopus, MEDLINE and Embase were conducted on the 18 July 2024, following a PICO approach. A flowchart, displaying the number of studies screened and included, can be found in Figure 1 and a full breakdown of the search strategy used can be found in Table 1. PROSPERO registration was not performed for this study.

The inclusion and exclusion criteria used to select the pertinent papers can be found in Table 2. Only studies in English language were considered.

The search results were saved and uploaded into the Covidence tool for screening. One study was found via citation searching and was added after the final search date [20]. Abstract and full-text screening was conducted by two reviewers (GC & DSCR), any conflicts were resolved by discussion. Data extraction was conducted by populating an Excel proforma with the columns determined through discussion and expert consultation concerning the key features for inclusion. Epidemiological, clinical, and radiological characteristics of the different patients were summarized, along with the surgical strategy used and the surgical outcomes in terms of extent of resection and postoperative deficits. The extent of resection was classified as gross total resection (GTR) when a macroscopically complete resection was performed, near total resection (NTR) was used when a resection >95% was performed, while when a residual tumour was present, the term subtotal resection (STR) was used.

The details on the targeted therapies used, their combination, posology, and duration were collected, along with the side effects and the radiological responses during treatment and at last follow-up. Radiological responses were classified according to volumetric analysis when reported, and at last follow-up they were divided into the following: complete response when the tumour was no more visible, near total response when a substantial reduction of tumour volume was reported (>80%), partial response when the reduction was between 80 and 20%, stable disease when no volumetric difference was noted, and progression when a volumetric increase was reported.

Meta-analysis was not possible due to the small number of studies found and the predominance of case-reports. Therefore, due to the qualitative nature of this review, risk of bias assessment was not deemed appropriate.

## 3. Results

**Figure 2:**
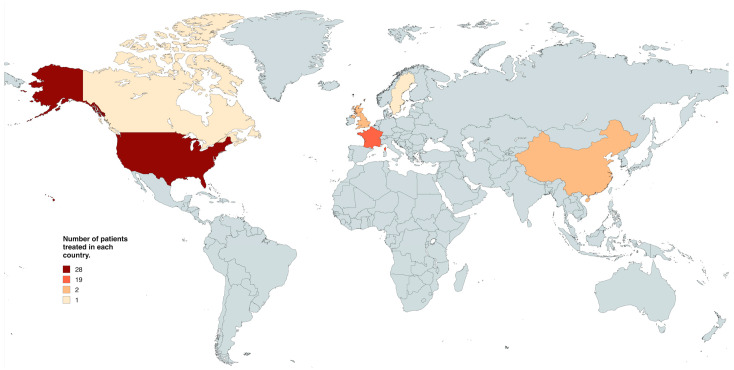
World map detailing the number of patients treated with targeted molecular therapy in each country.

The search yielded a total of 280 search results; 149 were duplicates, and 110 were excluded on title, abstract, and full-text screening. Full text screening yielded 21 studies for inclusion with 53 patients (Figure 1) [17,18,20,21,22,23,24,25,26,27,28,29,30,31,32,33,34,35,36,37,38]: 26 patients received BRAFi as adjuvant treatment, while 25 received them as neoadjuvant treatment. Two patients received BRAFi as palliative treatment. Almost all studies included case reports, with the exception of one study which was a case-series investigating the use of BRAF/MEK inhibitors as neoadjuvant treatment in 16 patients with papillary craniopharyngioma [18] and one cohort study composed of 16 patients using BRAFi as neoadjuvant, adjuvant, or palliative strategy [20]. Most patients were treated in the United States (n = 28, 52.8% Figure 2).

The median patient age in the adjuvant cohort was 46 years (IQR: 38.5–57.7), while in the neoadjuvant cohort, it ranged from 21 to 83 years. The literature cohort was constituted mostly by male patients (30/53, 56.6%). The most common clinical presentations were visual impairment (18/36, 50%), headaches, nausea, and vomiting (16/36, 44%). Partial anterior hypopituitarism was described in 19 out of 31 patients (61.3%), a stalk effect was reported in three patients and complete anterior hypopituitarism in one case, while diabetes insipidus (DI) was reported in 12 cases. The association of a solid and cystic morphology was the most common tumour feature (26/35, 70.3%), while isolated solid tumours were reported in nine cases. Most lesions were described as suprasellar and tuberoinfundibular, with six cases being confined to the third ventricle. Hypothalamic invasion was not systematically described but it was reported in 15 cases. One study reported a patient with a calcified tumour [20]. All the details are provided in Table 3 and Table 4.

Endoscopic transsphenoidal resection was reported in 9/18 cases (50%) receiving adjuvant BRAFi, while various craniotomies were used in the same number of cases, according to tumour extension. GTR and NTR were achieved in two cases each, while an STR was obtained in 13 cases (Table 5). In the neoadjuvant cohort, one study did not report the approach selected [35], while another did not perform any biopsy before starting the treatment with BRAFi [24] (Table 6). Postoperative outcomes were detailed in 10 cases belonging to the adjuvant cohort: in six patients new endocrinological deficits were described (Table 5), while one patient in the neoadjuvant cohort was complicated with panhypopituitarism and infarction in the territory of the anterior choroidal artery after tumoral biopsy [35].

In the adjuvant setting, BRAFi were used to treat recurrent tumours not responding to adjuvant radiation therapy [20,21,22,33,34,36,38], or they were introduced before the use of radiation therapy in some particular settings, dealing with young patients with limited or no endocrinological deficits or with tumours presenting a close contact with the optic apparatus [17,20,23,25,26,27,28,29,31,32,37]. In two cases BRAFi were used in a palliative setting, in patients experiencing recurrence or relevant residual tumours with failure of previous multimodal therapies [20].

The most common regimen included dabrafenib (BRAF Inhibitor) combined with Trametinib (MEK 1 and 2 inhibitor), reported in 13 out of 18 of case reports (72.2%) and on the cohort of De Alcubierre et al. [20]. Dabrafenib was also used as a stand-alone therapy in two cases [32,34], with one study using a higher dose of 225 mg twice daily [34]. Verumafenib, another BRAFi, was also used as a stand-alone therapeutic in two cases [28,38]. The details of the different protocols along with the corresponding radiological responses are reported in Table 7.

The mean duration of the adjuvant treatment with BRAFi was 8.8 months (median 5 months). There was a significant variability in therapy duration among the studies with treatment ranging from 52 days to 28 months, with 8 out of 25 patients (32%) still being under treatment at the moment of the reporting of their case. From a radiological point of view, during the treatment, a variable response from 24% to 100% of tumour volume reduction was observed at cerebral MRI. Globally, a reduction in tumour volume ≥ 80% (near total response) was described in 16 of 25 cases (64%) reporting volumetric analysis, while in 10 cases the reduction was ≥90%, involving both the solid and cystic portion of the tumour. Once the treatment was stopped (17 cases, 68%), adjuvant treatments were performed to stabilize patients in remission in 11 cases [17,20,27,29,31], or when a progression at follow up was detected, using surgery followed by radiation therapy [28], or a new cycle of BRAFi [25,38]. In three cases a stable disease was observed and no further therapies were administered [21,22,34]. Mean follow-up after the end of targeted therapy was 17.1 months (median 13.9). One patient died for tumour progression [25].

The neoadjuvant use of BRAFi was performed after a biopsy for histological confirmation of papillary craniopharyngioma and BRAF mutation in all the cases [17,20,27,35], while in one case surgery was refused by the patient and the treatment was started based on the empirical diagnosis of papillary craniopharyngioma on the cerebral MRI [24]. The details are reported in Table 8 and Table 9.

Nine patients (36%) were treated with a combination of dabrafenib and trametinib [24,27,35]. All the patients experienced a near complete response with this protocol, with 6 out of 8 having a tumour reduction ≥ 90% (75%) and the mean treatment duration was 5.7 months. Mean follow-up duration was 6.2 months for these patients and two patients were still on therapy, while two just finished the complementary radiation therapy. Brastianos et al. used a protocol combining verumafenib and cometinib in their cohort of 16 patients [17]. A near complete response was reported in 15 patients (94%), with a median tumour volume reduction between 85% and 91%. In one case no efficacy was recorded as the treatment was stopped early because of adverse events, while in three cases disease recurrence was observed once the treatment was stopped [18]. Ten out of 16 patients did not receive further treatments after the neoadjuvant protocol.

The most commonly reported side effects related to the treatment was pyrexia and cutaneous rashes. Pyrexia was probably related to the use of trametinib, as it ceased once the treatment was stopped [24]; it was not reported by Brastianos et al., as they used cometinib as MEK inhibitor [18]. Dabrafenib in monotherapy was associated with verrucal keratosis, that regressed when a MEK inhibitor was introduced [33]. CSF leak and pneumocephalus was reported in one case owing to rapid reduction in tumour volume due to treatment [38]. Some patients experienced toxic effects of treatment leading to posology reduction [22,28] or treatment cessation with resolution of adverse events [18,20,25].

## 4. Discussion

BRAF V600E mutation characterizes more than 90% of papillary craniopharyngiomas and this gain-of-function mutation leads to the persistent activation of the RAS/RAF/MEK/ERK cascade [8]. This pathway mediates cell proliferation, differentiation, and cell survival [39,40]. Furthermore, in papillary craniopharyngioma, BRAF V600E mutation could confer proliferative advantage to SOX2+ tumour cells [41,42].

This systematic review demonstrated early evidence of BRAFi combined with MEK inhibitors confers good control of papillary craniopharyngioma and favourable safety profiles. Therefore, BRAFi may represent an interesting strategy of treatment as *adjuvant treatment* at recurrence or tumoral progression or as *neoadjuvant treatment* to decrease tumour volume and allow the performance of potentially curative surgery or radiation therapy to increase the chances of achieving a long-lasting control of the disease. Indeed, multiple case reports and oncology reviews support the incorporation of adjuvant targeted therapy into the multimodal treatment approach for papillary craniopharyngioma, considered as a significant advancement in neuro-oncology [7]. On the other side, recent papers showed interesting radiological responses in patients undergoing neoadjuvant treatments with these targeted therapies [18,20]. Furthermore, its applications in palliative care may also represent a promising strategy where alterative multimodal strategies have failed [20].

*Adjuvant target therapy* was used in rapidly recurrent tumours or with tumours refractory to standard treatment modalities. Considering the aggressive nature of this subgroup of patients, the reported radiological response to treatment with BRAFi was largely positive. Indeed, BRAFi (dabrafenib and vemurafenib), alone or more frequently in combination with MEK inhibitors (trametinib and cobimetinib), showed encouraging results with ≥80% of reduction of tumour volume in more than 60% of reported cases. Authors mainly administered a combined drug regimen as it demonstrated superior oncological outcomes, compared to BRAFi monotherapy, in BRAF V600E-mutant melanoma [15,16]. According to our analysis, monotherapy as adjuvant treatment allowed adequate tumour control in two cases [32,34], while progression was observed in one case [28]. Although this does not provide conclusive evidence that combination therapy has a greater efficacy, it supports that dual BRAF and MEK inhibition should be the first treatment option in patients without contraindications to treatment. The points requiring clarification are the posology used along with the duration of therapy, as it largely varied among studies from some weeks to more than 2 years of ongoing treatment. Relapse after treatment cessation was reported in three cases [25,28,38], with a new response once the treatment was re-started. This may indicate a subset of patients with BRAFi dependent lesions requiring further definitive management, or long-term treatment if they remain poor candidates for surgery and radiotherapy. The follow-up, to assess the radiological response once the treatment was stopped, was therefore also heterogeneous, varying from patients still under treatment at the moment of reporting their case to long follow-up of 48 months. Long-term follow-up will provide essential insight into the tumour behaviour following treatment cessation and whether BRAF and MEK inhibitor resistance can be explained by novel tumour genetic adaptations. In those cases, further definitive management would be required if clinically appropriate while tumours developing resistance to adjuvant BRAFi treatment would require further investigation. Future strategies may make use of alternative BRAFi or require the development of novel target therapies.

In addition to three case reports, the efficacy of combining BRAF and MEK inhibitors in a *neoadjuvant regimen* was confirmed in a phase-2 clinical trial (NCT03224767) including 16 patients with newly diagnosed papillary craniopharyngiomas, treated in 28-day cycles (Table 9) [18] and in a recent cohort of 6 patients [20]. In the article of Brastianos et al. the mean volume reduction of the tumour was 91%, thus supporting their upfront administration after a biopsy/subtotal surgical resection, with the goal of reducing tumour volume and thus the rate of postoperative complications, while also limiting the dose of radiation therapy administered [18]. Similarly, if we summarize the other reports, 75% of patients showed a tumour reduction ≥ 90%. Some authors also propose the use of neoadjuvant BRAF/MEK inhibitors in patients with neurological deficit, to benefit from the rapid tumour shrinkage [35].The timing of administration should be tailored to each patients’ characteristics. The pitfall in the use of BRAFi for papillary craniopharyngioma is the necessity of performing an initial biopsy to assess histopathology and BRAF mutation. As for other CNS tumours, further advances in deep-learning radiomics analysis of craniopharyngiomas could help in the future in avoiding biopsies, predicting the presence of BRAF mutation before surgery and thus favouring the use of targeted therapies as a neoadjuvant regimen [43,44,45]. Papillary craniopharyngiomas generally present as intrasellar or isolated intraventricular lesions, with well-defined margins, and they are predominantly solid, with hypointense microcystic portions on T1-weighted images while calcifications are rare [46]. The model proposed by Cheng et al. showed excellent results as it could differentiate between adamantinomatous and papillary craniopharyngiomas with an AUC of 0.96 and an ability to differentiate BRAF V600E mutation from wild type craniopharyngiomas with an AUC of 0.92 [45]. These analyses could thus facilitate non-invasive estimation of pathological subtypes and genetic mutational status, allowing for neoadjuvant treatment without biopsy. The other alternative would be to perform a genetic sequencing in a peripheral blood test, but this technique remains expensive as it requires complex laboratory settings, and results are currently controversial [17,47,48].

Globally, these targeted therapies seem to be associated with a favourable risk profile [18]. Nevertheless, their use should be standardized and included into a larger multidisciplinary approach, as their applications are not curative and require further strategies of treatment to control the disease. New clinical trials using BRAFi may solidify the use of target therapies as a robustly evidenced therapeutic tool [49,50,51,52], with standardized protocols of treatment. Further identification of patient or tumour-related factors may help in defining the inclusion criteria for the three emergent treatment strategies, namely the neoadjuvant, adjuvant, and palliative applications of BRAFi [18,20].

Despite these advancements in the management of papillary craniopharyngiomas, limited progress has been made in the management of adamantinomatous craniopharyngiomas, where a range of different molecular therapies have been employed (anti-EGFR, anti-IL6 and anti-VEGF) with heterogenous results [53,54,55]. The treatment of adamantinomatous tumours remains a point of contention in the literature and clinical practice. Nevertheless, the recent progresses made in the management of papillary craniopharyngioma will motivate future research effort to discover novel treatment strategies for adamantinomatous craniopharyngioma through molecular and genetic studies.

## 5. Conclusions

The treatment of papillary craniopharyngioma is shifting from the neurosurgical and endocrinological field to a more complex multidisciplinary management, including radiation therapists, pathologists, and oncologists. Adjuvant and neoadjuvant applications of BRAF and MEK inhibitors showed exciting results, opening new treatment avenues, particularly for recurrent tumours and for patients who are poor surgical and radiotherapy candidates, offering them an opportunity to reduce treatment related morbidity. The current promise should be confirmed in large scale comparative trials to approve the BRAFi protocol of use.

## Figures and Tables

**Table 1:** The search strings used in each database are detailed here.

Database	Search String
Scopus	TITLE-ABS-KEY (craniopharyng*) AND TITLE-ABS-KEY (mole* OR targe* OR braf OR dabrafenib OR trametini) AND TITLE-ABS-KEY (adju* OR neoadj*)
PubMed	Craniopharyng* and (adju* or neoadj*) and (molec* or targe* or BRAF or dabrafenib or trametinib))
MEDLINE and Embase	(craniopharyng*) AND (targeted therapy OR molecular therapy OR BRAF OR dabrafenib OR trametinib) AND (adju* OR neoadj*)

**Table 2:** The inclusion and exclusion criteria used for screening the pertinent articles included in the analysis.

Inclusion	Exclusion
Clinical reports and conference abstracts	Pre-clinical studies
Neoadjuvant use of BRAF inhibitors	Non-English studies
Adjuvant use of BRAF inhibitors	Studies reporting patients with adamantinomatous craniopharyngioma
Studies including papillary craniopharyngiomas	

**Table 3:** Summary of epidemiology data, clinical and radiological features of patients treated with adjuvant BRAF inhibitors.

Study	Age (Years)	Sex	Comorbidities	Clinical Presentation	Pituitary Hormone Deficiency	MRI Finding	Solid/Cystic Morphology
Brastianos 2015 [17]	39	M	None	Headache and confusion	None	Solid sellar and suprasellar enhancing tumour	Solid-cystic
Aylwin 2016 [38]	57	F	ns	Visual impairment	Hyperprolactinaemia	Sellar and suprasellar mass with perifocal oedema	Solid
Rostami 2017 [37]	65	M	Ancient history of sine materia SAH	Nausea and weight loss bitemporal hemianopia	Hypocorticism Hypothyroidism	Sellar and suprasellar lesion with cystic components	Solid-cystic
Roque 2017 [36]	47	F	ns	headache Visual impairment, amenorrhea, cold intolerance	Hypogonadism and hypothyroidism	Cystic lesion with nodular enhancement, suprasellar and infiltrating the floor of 3rd ventricle	Solid-cystic
Himes 2019 [34]	52	M	Non-Hodgkin lymphoma and stage III colon cancer	Visual impairment polydipsia, polyuria	DI	Suprasellar lesion	Solid-cystic
Bernstein 2019 [33]	60	M	ns	ns	ns	ns	ns
Rao 2019 [32]	35	M	None	Headaches, nausea, and vomiting Short-term memory loss	None	Third ventricular mass obstructing foramen of Monro with obstructive hydrocephalus	Solid-cystic
Khaddour 2020 [31]	39	M	None	Headache bitemporal hemianopsia	None	Homogenous enhancing suprasellar lesion	Solid
Gopal 2020 [30]	44	M	ns	Fatigue, weight gain, polydipsia, polyuria	Hypogonadism DI	Solid-cystic suprasellar mass	Solid-cystic
Di Stefano 2020 [29]	55	F	None	Weight gain	Hypopituitarism	Suprasellar mass	Solid-cystic
Chik 2021 [28]	37	M	Recurrent sinusitis Obesity	Visual impairment and headaches	Complete anterior hypopituitarism	Enhancing sellar and suprasellar mass	Solid
Calvanese 2022 [27] (1)	40	M	None	Bitemporal inferior quadranopia	Hypogonadism	Suprasellar and tubero-infundibular lesion infiltrating the floor of the 3rd ventricle	Solid-cystic
Nussbaum 2022 [26]	35	M	ns	Confusion and memory loss	ns	Suprasellar solid and cystic mass	Solid-cystic
Wu 2023 [25]	63	F	ns	Visual impairment	ns	Sellar and suprasellar enhancing mass	Solid
Wu 2023 [25]	75	M	ns	Headaches and dizziness	ns	Sellar and suprasellar enhancing mass with a cystic portion	Solid-cystic
Yu 2024 [23]	45	M	None	Headache	None	Homogenous enhancing mass, intraventricular with hydrocephalus	Solid
Butt 2020 [22]	32	F	Basal cell carcinoma	Visual impairment	ns	Suprasellar mass	ns
Shah 2023 [21]	57	F	None	Visual impairment, headaches, and nausea	Hyperprolactinemia	Enhancing suprasellar mass	Solid-cystic
De Alcubierre 2024 [20] (adjuvant: 8 patients and palliative protocol: 2 patients)	Mean 43.5	4 M, 6 F	1 colorectal cancer in remission	4 headaches 5 visual impairment	9 anterior hypopituitarism 8 posterior hypopituitarism 7 panhypituitarism 3 hypothalamic symptoms	4 suprasellar lesions 1 with secondary ventricular invasion 3 infundibulo-tuberal 1 intraventricular 6 lesions with hypothalamic invasion 1 calcified	1 solid lesion 9 solid-cystic

Abbreviations: F: female; M: male; ns: not specified; DI: Diabetes Insipidus; SAH: Subarachnoid haemorrhage.

**Table 4:** Summary of epidemiology data, clinical and radiological features of patients treated with neoadjuvant BRAF inhibitors.

Study	Age (Years)	Sex	Comorbidities	Presentation	Pituitary Hormone Deficiency	MRI Finding	Solid/Cystic Morphology
Juratli 2019 [35]	21	M	ns	Headaches and fatigue Weight gain Nausea Visual field deficits	ns	Enhancing suprasellar mass	Solid-cystic
Calvanese 2022 [27]	69	M	HIV	Right visual impairment and psychiatric changes	Hyperprolactinaemia	Solid infundibular lesion, invading the floor of the 3rd ventricle	Solid
Lin 2023 [24]	59	M	None	Headache	None	Mixed suprasellar nodular and cystic lesion	Solid-cystic
Brastianos 2023 [18]	Age Range 33–83 years	7 M, 7 F	ns	ns	ns	ns	ns
De Alcubierre 2024 [20] (neoadjuvant protocol)	Mean 60.3	4 M, 2 F	1 HIV 1 multiple sclerosis	1 headache 4 visual impairment	5 anterior hypopituitarism 2 posterior hypopituitarism 2 panhypituitarism 3 hypothalamic symptoms	5 infundibulo-tuberal lesions, with hypothalamic invasion 1 purely intraventricular	2 solid, 4 solid-cystic

Abbreviations: F: female; M: male; ns: not specified.

**Table 5:** Summary of the clinical management and surgical outcomes in patients receiving BRAF inhibitors as adjuvant treatment.

Study	Surgical Approach	Extent of Resection	Adjuvant Radiation Therapy before BRAFi	Time to Recurrence	Genetic Profile	Post-Operative Deficit
Brastianos 2015 [17]	Craniotomy	STR	N (Administered after BRAFi 50.4 Gy in 28 fractions)	7 months from 1st surgery then rapid regrowth with emergency decompressions every 2–4 weeks	BRAF V600E	DI, central hypothyroidism and secondary adrenal insufficiency
Aylwin 2016 [38]	Endoscopic transsphenoidal	STR	Y (dose ns)	4 years → surgery and then BRAFi	BRAF V600E	ns
Rostami 2017 [37]	Endoscopic transsphenoidal	STR	N	3 weeks	BRAF V600E	ns
Roque 2017 [36]	Frontal craniotomy	STR	Y (54 Gy in 30 fractions)	1 month → Radiation therapy 4 months after radiation therapy → BRAFi	BRAF V600E	DI and central adrenal insufficiency
Himes 2019 [34]	Pterional craniotomy	STR	Y (36 Gy in 12 fractions)	3 years	BRAF V600E	Panhypopituitarism
Bernstein 2019 [33]	Endoscopic transsphenoidal	ns	Y (dose ns)	ns	BRAF V600E	ns
Rao 2019 [32]	Craniotomy	STR	N	ns	BRAF V600E	DI, central hypothyroidism and central adrenal insufficiency
Khaddour 2020 [31]	Endoscopic transsphenoidal	NTR	N	5 months	BRAF V600E	None
Gopal 2020 [30]	Craniotomy	STR	N	NS	BRAF V600E	ns
Di Stefano 2020 [29]	Endoscopic transsphenoidal	STR	N	3 months	BRAF V600E	ns
Chik 2021 [28]	Endoscopic transsphenoidal	GTR	N (Administered after BRAFi 50 Gy in 30 fractions)	6 weeks from last surgery (3 surgeries in childhood)	BRAF V600E	None
Calvanese 2022 [27]	Endoscopic transsphenoidal	NTR	N	8 months	BRAF V600E	DI and central hypothyroidism
Nussbaum 2022 [26]	Bifrontal craniotomy	STR	N	ns	BRAF V600E	DI and central hypothyroidism
Wu 2023 [25]	Bifrontal craniotomy	STR	N	3 months	BRAF V600E	ns
Wu 2023 [25]	Endoscopic transsphenoidal	GTR	N	15 months from the 1st surgery and then 2 months after the 2nd	BRAF V600E	ns
Yu 2024 [23]	Transventricular	STR	N	4 months	BRAF V600E	None
Butt 2020 [22]	Craniotomy	STR	Y (dose ns)	2 months after radiation therapy	BRAF V600E	ns
Shah 2023 [21]	Endoscopic transsphenoidal	STR	Y (54 Gy in 30 fractions)	3 months → 2nd surgery Growth of cystic portion during radiation therapy	BRAF V600E	Anterior hypopituitarism Blindness during adjuvant radiation therapy
De Alcubierre 2024 [20] (adjuvant and palliative protocol)	ns	ns	Only 1/8 cases	ns	BRAF V600E	ns

Abbreviations: DI: Diabetes Insipidus; ns: Not Specified; STR: Subtotal Resection; GTR: Gross-total Resection; Gy: Gray’s.

**Table 6:** Summary of the clinical management in patients receiving BRAF inhibitors as neoadjuvant treatment.

Study	Initial Surgical Approach	Aim of Resection	Genetic Profile	Post-Operative Deficit
Juratli 2019 [35]	Surgery	Biopsy	BRAF V600E	Panhypopituitarism Infarction in anterior choroidal artery territory
Calvanese 2022 [27]	Transventricular	Biopsy	BRAF V600E	None
Lin 2023 [24]	None	Not performed	Blood sample, Negative for BRAF mutation	Not described

**Table 7:** A summary of the targeted therapy and treatment regimens used in an adjuvant fashion in the different case reports published in literature, along with the radiological responses and follow-up periods.

Study	Timing after Surgery	Therapeutic (1)	Therapeutic (2)	Duration	Adverse Events	Tumour Reduction	Total Follow-Up	Ongoing BRAFi Therapy	Radiological Follow-Up
Brastianos 2015 [17]	7 weeks after last surgery	Dabrafenib (150 mg twice daily)	Trametinib (2 mg once daily)	52 days	None	85% by volume	7 months	N	Near complete response after BRAFi followed by: New surgery for removal of residual tumour Adjuvant radiation therapy
Aylwin 2016 [38]	Some weeks after 2nd surgery	Vemurafenib (960 mg twice daily)		3 months	CSF leak, pneumocephalus and meningitis	Near complete	7 months	N	Initial near complete response (under BRAFi) → recurrence after 6w of pause → new response to BRAFi → new progression at 7 months (BRAFi stopped)
Rostami 2017 [37]	3 weeks	Dabrafenib (150 mg twice daily)	Trametinib (2 mg once daily)	15 weeks	Pyrexia	91% by volume	15 weeks	Y	Near complete response Still under treatment
Roque 2017 [36]	4 months after radiation therapy	Dabrafenib (150 mg twice daily)	Trametinb (2 mg once daily)	7 months	Pyrexia	75% by volume	7 months	Y	Partial response Still under treatment
Himes 2019 [34]	5-years post-surgery	Dabrafenib (225 mg twice daily)		12 months	Joint pain	Near complete response starting 6 months after beginning of the treatment Dose affected by AE	24 months	N	Near complete response Stable at 1 year
Bernstein 2019 [33]	ns	Dabrafenib (150 mg twice daily)	Trametinib (2 mg once daily)	28 months	Diffuse verrucal keratosis under dabrafenib alone	100% by tumour volume	28 months	Y	Complete response Still under treatment
Rao 2019 [32]	ns	Dabrafenib (150 mg twice daily)		24 months	None	Partial response at 2 months and near complete response at 1 year	24 months	Y	Near complete response Still under treatment
Khaddour 2020 [31]	1 week post recurrence	Dabrafenib (150 mg twice daily)	Trametinib (2 mg once daily)	9 months	Pyrexia	70% by volume	26 months	N	Partial response Stable at 2 years GK performed during follow-up
Gopal 2020 [30]	ns	Dabrafenib	Trametinib	ns	ns	ns	ns	ns	Partial regression
Di Stefano 2020 [29]	5 months post-surgery	Dabrafenib (150 mg twice daily)	Trametinib (2 mg once daily)	30 weeks	Fatigue, cough and peripheral oedema	95% by volume	55 weeks	N	Near complete response Stable at 6 months PBRT performed after 30 weeks of BRAFi
Chik 2021 [28]	60 days post-surgery	Vemurafenib (960 mg twice daily)		25 months	Arthralgia, myalgia, photosensitivity, and elevated liver enzymes Dose of vemurafenib was reduced	55% by volume	25 months	Y	Progression of cystic portion after 8 months of treatment → surgery and RTH 17 months after RTH: new growth of cystic component → GK Still under treatment
Calvanese 2022 [27]	8 months post-surgery on tumour recurrence	Dabrafenib (150 mg twice daily)	Trametinib (2 mg once daily)	5 months	None	90% by volume	14 months	N	Near complete response Stable at 14 months Radiation therapy at the end of BRAFi
Nussbaum 2022 [26]	2 months post-surgery	Dabrafenib (150 mg twice daily)	Trametinib (2 mg once daily)	22 months	Anemia and elevated liver enzymes	95% by volume	22 months	Y	Near complete response Still under treatment
Wu 2023 [25]	3 months post-surgery	Dabrafenib (150 mg twice daily)	Trametinib (2 mg once daily)	3 months	None	>95% by volume	24 months	N	Progression at 2 years follow-up → new start of BRAFi with regression of the solid component
Wu 2023 [25]	5 months post-surgery	Dabrafenib (150 mg twice daily)	Trametinib (2 mg once daily)	3 months	Hyperglycaemia and lower limb oedema → BRAFi stopped	24% by volume	6 months	N	Tumour progression → death
Yu 2024 [23]	3 weeks post-recurrence	Vemurafenib (960 mg twice daily)	Cobimetinib (60 mg once daily) for cycles of 21 days	2 months	Diarrhoea, nausea and hypertension	98% by volume	29 months	N	Near complete response Stable at 29 months
Butt 2020 [22]	2 months post-recurrence	Dabrafenib (150 mg twice daily)	Trametinib (2 mg once daily)	3 months	Pyrexia and rash Dose reduction	ns “stable disease”	3 months	Y	Stable appearance Still under treatment
Shah 2023 [21]	4 months post-recurrence	Dabrafenib	Trametinib	ns 1 month probably	Rash and fatigue Prone to infectious diseases	>95% by tumour volume	4 years	N	Near complete response Stable at 4 years

Abbreviations: AE: adverse events; BRAFi: BRAF inhibitor; GK: Gamma Knife; ns: not specified; RTH: Radiotherapy.

**Table 8:** A summary of the targeted therapy and treatment regimens used in a neoadjuvant fashion in the different case reports, along with the radiological responses and follow-up periods.

Study	Timing	Therapeutic (1)	Therapeutic (2)	Duration	Adverse Events	Tumour Reduction	Total Follow-Up (Post-Chemotherapy)	Radiological Follow-Up
Juratli 2019 [35]	ns	Dabrafenib (150 mg twice daily)	Trametinib (2 mg once daily)	ns	ns	85% by volume	6 months	Near complete response
Calvanese 2022 [27]	3 months post-diagnosis	Dabrafenib (150 mg twice daily)	Trametinib (2 mg once daily)	4 months	None	90% by volume	2 months	Near complete response
Lin 2023 [24]	5 months post-diagnosis	Dabrafenib (150 mg twice daily) Then maintenance dose 75 mg twice daily	Trametinib (2 mg once daily)	6.5 months, still ongoing	Pyrexia Trametinib was stopped Atrial flutter (association not clear)	ns	On therapy	Near complete response

Abbreviations: ns: not specified.

**Table 9:** Summary of the clinical results of the cohort studies reporting the use of BRAF inhibitors for the treatment of papillary craniopharyngioma.

Study	Age and Sex	Extent of Resection	Type of Protocol	Therapeutic (1)	Therapeutic (2)	Duration	Adverse Events	Tumour Reduction	Total Follow-Up (Post-Chemotherapy)	Radiological Follow-Up
Brastianos 2023 [18]	Age Range 33–83 years, 7 male	Biopsy or subtotal	Neoadjuvant	Vemurafenib (960 mg twice daily)	Cobimetinib (60 mg once daily)	28-day cycles (Vemurafenib 28 days and cobimetinib 21 days), median number of 8 cycles	12 patients experienced either a rash, dehydration, ALP rise or QTc prolongation. One asymptomatic rise in CK. One hyperglycaemia 3 discontinued TT	91% median volume reduction 15 had complete or near complete response. 1 non-responder stopped treatment after 8 days due to adverse event	Median 22 months (95% CI 9–19)	Three disease progression once therapy was stopped 7 patients received no treatment after the protocol 6 RTH 1 RTH + surgery 1 RTH + dabrafenib 1 off-protocol vemurafenib–cobimetinib
De Alcubierre 2024 [20]	Mean age 50.5 years, ±15; 8 male	6 biopsy, 10 previously attempted radical surgery	6 neoadjuvant 8 adjuvant 2 palliative	Dabrafenib (150 mg twice daily)	Trametinib (1 or 2 mg once daily)	Mean duration: 5.8 months in the neoadjuvant setting; 7.5 months in in the adjuvant setting; 18.5 months in the palliative setting	2 patients increased liver enzymes, 1 myalgia, 1 vomiting and fever, 1 fatigue and peripheral oedema, 1 pneumopathy	81.4% mean reduction at last follow-up: Mean reduction of 89% with the neoadjuvant protocol; 73% with the adjuvant protocol; 91% in the palliative setting	Follow-up available only for 10 patients (4 patients still ongoing TT) Neoadjuvant TT: mean 11 months Adjuvant TT: mean 9 months Mean of 11.5 months (10 patients)	6/6 near total response in neoadjuvant protocol (>80% of tumour reduction); 5 received RTH In the adjuvant protocol: 4/8 near total, 3 partial response, 1 stable disease; 7 received RTH In the palliative protocol: 2/2 near total response

Abbreviations: CK: Creatine Kinase; RTH: Radiotherapy; TT: Targeted Therapy.

## Data Availability

Data sharing is not applicable. No new data were created or analyzed in this study.
